# Rubella sero-prevalence among children in Kilimanjaro region: a community based study prior to the introduction of rubella vaccine in Tanzania

**DOI:** 10.1186/s13052-017-0379-3

**Published:** 2017-07-21

**Authors:** Nikolas A. S. Chotta, Melina Mgongo, Jacqueline G. Uriyo, Sia E. Msuya, Babill Stray-Pedersen, Arne Stray-Pedersen

**Affiliations:** 1 Institute of Clinical Medicine, University of Oslo, Oslo, Norway; 2 Better Health for African Mother and Child, Moshi, Tanzania; 3 Institute of Public Health, Department of forensic medicine, University of Oslo, Oslo, Norway; 4 Division of Gynaecology and Obstetrics, Oslo University Hospital, Rikshospitalet, Oslo, Norway; 5 Institute of Public Health, Department of Community Health, Kilimanjaro Christian Medical University College, Moshi, Tanzania; 6 Institute of Public Health, Department of Epidemiology and Biostatistics, Kilimanjaro Christian Medical University College, Moshi, Tanzania; 7 P.O. Box 8418, Moshi, Tanzania

**Keywords:** Childhood, ELISA, Immunity, Prevalence, Rubella, Vaccination

## Abstract

**Background:**

Childhood rubella infection is a mild, self-limiting illness. Rubella infection among pregnant women however, is a major public health concern. Depending on gestation age, it may result in fetal death, stillbirth or a new-born with congenital rubella syndrome (CRS). Maternal antibodies protect young infants from rubella infection and lifelong immunity is acquired by vaccination or post-rubella infection. This study aims at characterizing rubella infection and its epidemiology in the Kilimanjaro region, prior to the introduction of the rubella vaccine in Tanzania.

**Methods:**

This was a population based cross-sectional study, covering all the seven districts in Kilimanjaro region, North-eastern Tanzania. The study population included children of 0 to 36 months of age and their mothers/primary caretakers. A multistage sampling method was used to obtain a representative sample of the children. Interviews were conducted using a structured questionnaire. Dried blood spot (DBS) samples were taken from eligible children. Rubella specific IgG antibodies were detected from eluted serum by enzyme-linked immunosorbent assay (ELISA). Descriptive statistics were used to summarize the data, the difference between groups was tested by Fishers exact test or chi square test as appropriate. Univariate and multivariate analysis was used, with rubella sero-positive groups as dependent variables and the socio-demographic, children, paediatric and parental factors as independent variables, the Odds ratio and their 95% confidence intervals were calculated to assess the strength of association between the dependent and independent variables. A *p* value less than 0.05 was considered significant.

**Results:**

The overall rubella sero-prevalence was 1.8%. Rural residence was associated with greater risk for rubella infection. Other family characteristic did not predict rubella infection.

**Conclusions:**

This study highlights the low natural immunity to rubella among children prior to the introduction of rubella vaccine in Tanzania. Our research underscores the need for an effective rubella vaccination program to prevent CRS. More epidemiologic and immunologic studies are needed to guide the vaccination deployment and administration strategy in Tanzania.

**Electronic supplementary material:**

The online version of this article (doi:10.1186/s13052-017-0379-3) contains supplementary material, which is available to authorized users.

## Background

Rubella is a mild and self-limiting illness [1]. The causative agent of rubella is the rubella virus, a single stranded RNA virus of the genus Rubivirus in the Togaviridae family. Rubella virus maintains only one serotype exclusively able to affect humans [2]. Transmission can occur through respiratory droplets or direct contact. Vertical transmission of rubella virus is also possible [1].

Congenital rubella infection has been shown to cause the most severe form of rubella disease. This route of transmission results from maternal rubella infection before conception or in early months of pregnancy. Depending upon the timing of fetal infection, infection may result in loss of pregnancy or congenital rubella syndrome (CRS) [1, 3].

Before the development of the rubella vaccine, rubella infection was prevalent worldwide. Outbreaks occurred in cycles every 5–9 years [1]. In countries where rubella vaccination campaigns have not yet begun or where administration is currently sub-optimal, rubella outbreaks continue to expose susceptible women to an increased risk for miscarriages, still births or CRS in their newborns [4–8]. Population susceptibility to infection is predicted by herd immunity, population density, place of residence, socio-economic factors, as well as other epidemiologic indicators [9–12].

Prevention of rubella is aimed at reducing the risk of congenital infection and consequent CRS [1, 3, 12]. Recent research has recommended that inclusion of rubella containing vaccines (RCVs) in universal childhood vaccination programs, could be targeted as a major prevention strategy [10]. Other available prevention strategies include: routine childhood vaccination, mass vaccination of adolescents and adults, or a combined strategy [13–15]. In order to select an optimal, evidence-based vaccination strategy for a particular geographic region, further information regarding the epidemiology of rubella infection is needed, along with a better understanding of desired outcomes (control or elimination) and availability of resources [16].

Rubella vaccines are available in monovalent or in combined forms like; Measles and Rubella (MR), Measles, Mumps and Rubella (MMR), Measles, Mumps, Rubella and Varicella (MMRV), the vaccines are safe and effective, with rubella sero-conversion rates of >95% after a single dose [16–18]. To be effective, rubella vaccination coverage must be high enough to achieve herd immunity. Vaccination coverage of less than 80% may result in a shift of infection to young adults, with resultant increase in CRS [5, 19]. Routine and high vaccination coverage has successfully eliminated rubella in most North American and European countries [20, 21].

Most epidemiologic data on rubella infection are derived from ongoing Measles surveillance. Given that rubella is a mild disease and asymptomatic in up to 50% of cases, rubella infection and associated CRS is oftentimes missed and/or under-reported.

In Tanzania, though vaccination against rubella was started in 2014 (Additional file 1), there are few studies describing the epidemiology of rubella or the efficacy of interventions [22–24]. More epidemiologic studies are needed to estimate gross disease burden, and explore disease distribution along with associated risk factors for rubella infection. This information could provide the evidence base to better inform the planning, monitoring and evaluation of rubella prevention program [22, 25].

Our study aims to determine the pre-vaccination rubella sero-prevalence among children in the Kilimanjaro region of Tanzania [26, 27]. Predictors of rubella infection including: age, maternal age, parity, urban versus rural settings and other socio-economic factors were explored [28]. Our findings will provide pilot data for future large-scale rubella studies, and aid in the planning for immediate public health campaigns as well as long-term rubella elimination policy [29].

## Methods

### Study design and area

The study was a community based cross-sectional study, covering all the seven districts of the Kilimanjaro region in North-eastern Tanzania. Kilimanjaro is among 30 administrative regions of Tanzania, and covers an area of 1831.32 km^2^. Kilimanjaro maintains an estimated population of 1,640,087, of those, 451,911 of which are women of the reproductive age. The fertility rate (per woman) is 4.8, with an annual growth rate of 1.6%. In Kilimanjaro, there are 152,198 children aged 0–36 months. Most residents (up to 80%) engage in subsistence farming and live in rural areas [30].

### Study population

The study population included children of 0 to 36 months of age and their mothers/primary caretakers, in seven districts of the Kilimanjaro region. The other inclusion criteria was presence of blood sample for rubella testing. The specific exclusion criteria was; children with proven vaccination with a rubella containing vaccine, were not eligible for this study..

### Sampling method

From June 2010 to May 2011, a multistage sampling method was used to obtain a representative sample of children 0 to 36 months [31]. Sampling was first conducted by a random cluster sampling method, and fifty clusters were selected from 7 districts proportionate to the population size. This was achieved from a sampling frame, which included the number of all children 0 to 36 months in each village, with a column for cumulative population. The total population was divided by the number of clusters to get the sampling interval of 166,808/50. The first cluster was determined by multiplying a computer generated random number between 0 and 1 and the sampling interval. By adding the previous number to the sampling interval, the subsequent clusters were located, to the total of fifty clusters. A compact segment sampling method was applied to select fifty children 0 to 36 months in each cluster. The selected clusters were mapped into segments of fifty children, each segment was allocated a number on a piece of paper, and one was selected randomly. In each selected segment, a quota sampling method was used for the children to be recruited. Recruitment was done by visiting all households in the segment until fifty children were enrolled. In the case that fewer than fifty eligible children were enrolled in a segment, enrollment went to a second randomly selected segment. To maximize recruitment, prior information was given to mothers/caretakers and their eligible children to be available on the day of interview.

### Interviews

Interviews were conducted using a structured questionnaire. Information collected included: social, economic and demographic characteristics, such as age, sex, maternal education level, marital status, occupation, income, household facilities, reproductive history, pediatric and child health history.

### Blood sample collection and laboratory methods

Dried blood spots (DBS) were taken from eligible children by finger or heel prick. Blood was dropped onto standardized filter papers, and a corresponding participant identification number was written on the sample. The filter papers were air dried and sealed in airtight plastic bags. The samples were sent to the Kilimanjaro Christian Medical Centre (KCMC) Clinical laboratory, where they were stored in a minus 20 degree Celsius freezer prior to analysis. Rubella specific IgG antibodies were detected from eluted serum by enzyme-linked immunosorbent assay (ELISA), using commercial test kits from Human Diagnostics worldwide, HUMAN Gesellschaft für Biochemica und Diagnostica mbH, Germany (ref. 51,208). The presence of rubella-specific IgG was determined by comparing the optical density (OD) to the cut off ranges (index). A positive test was determined when the absorbance test result at 450 nm was ≥0.8. For quantitative estimation of rubella IgG in positive test specimens, the OD was converted to International Units per milliliter by plotting a standard curve for each assay according to the test kit instructions. The estimated levels were read off the graph using their individual A450. A positive test was defined as a titer equal or more than 15UI/ml, a negative result was defined as a titer ≤10 UI/ml. Equivocal results were defined as a titer of 10–15 UI/ml, indeterminate specimens were retested until test results were conclusive.

### Data management

Data obtained from questionnaires and laboratory test results were entered and verified for consistency. Statistical analysis was performed using the Statistical Package for Social Sciences (SPSS) for windows version 16. Rubella infection status was assigned as the dependent variable, and all other epidemiologic indicators were assigned as the independent variables. Descriptive statistics were used to summarize the data. Results are expressed as real numbers and percentages were assigned to rubella sero-prevalence results. Differences between groups were compared using Fishers exact test or chi square test as appropriate. Univariate and multivariate analysis were used, with rubella sero-positive groups assigned as dependent variables and epidemiologic factors assigned as independent variables. Odds ratios, along with their 95% confidence intervals, were calculated to assess the strength of association between the dependent and independent variables. A *p*-value of <0.05 was considered statistically significant.

## Results

A total of 1870 children (0 to 36 months) were enrolled and provided blood samples tested for rubella. The majority (93.6%) of patient enrolled were 7–36 months. The 1751 (93.6%) of the children in the 7–36 months group, were included in this analysis. The mean age of the participants was 15.9 months (SD = 6.5), and the male enrollment rate was 902/1751 (51.5%). Children were enrolled from all districts of Kilimanjaro region, and their residence statuses were classified as 25.8% urban and 74.2% rural. The children’s mean hemoglobin level was 10.2 (SD = 1.5), and 97% of them had complete vaccination status for their age group.

The interviewed mother’s mean age was 28.7 (SD = 7) years. Many of the participant mothers (48.8%) were peasant farmers. Antenatal attendance was 98.2%, delivery in medical facility was 86.8% and 95.2% of mothers practiced breast feeding on demand.

Rubella sero-prevalence among participant children was 1.8% (32/1751). Rubella sero-prevalence increased with age from 1.1% in the 7 to12 month age group, to 2.3% in 13 to 24 month and 25 to 36 month age groups.

We observed that, rubella sero-prevalence varied among the districts. Same district demonstrated the highest sero-prevalence (5.1%) and Moshi rural district maintained the lowest sero-prevalence (1.2%) (*p* = 0.001). Children in Rombo and Moshi districts had lower sero-prevalence compared to those in Same district (OR 0.3, 95% CI: 0.1, 0.8) for Rombo, and (OR 0.2, 95% CI: 0.1, 06) for Moshi district. Children of Mothers doing small business/unskilled jobs had lower rubella sero-prevalence as compared to those of skilled/professional’s (OR 0.1, 95% CI: 0.02, 0.7). We observed no positive rubella test results from individuals sampled from Hai, Siha and Moshi urban districts (Table 1).

**Table 1:** Background characteristics and rubella seropositivity, *N* = 1751

Variable name	N	%	Rubella sero-positive % (n)	Chi- square *p*-value
Total	1751	100	1.8 (32)	
Child’s gender				
Male	902	51.5	1.9 (17)	0.5
Female	849	48.5	1.8 (15)	
Child’s age categories				
7–12 months	647	37	1.1 (7)	0.182
13–24 months	932	53.2	2.3 (21)	
25–36 months	172	9.8	2.3 (4)	
Child’s District of residence				
Same	312	17.8	5.4 (17)	< 0.001
Mwanga	132	7.5	2.3 (3)	
Rombo	359	20.5	1.7 (6)	
Moshi District	448	25.6	1.3 (6)	
Moshi municipal	180	10.3	-	
Hai	172	9.8	-	
Siha	148	8.5	-	
Child’s Residence (*N* = 1727)				
Rural	1282	74.2	2.2 (28)	0.018
Urban	445	25.8	0.7 (3)	
Mother’s occupation^a^ (*N* = 1690)				
Unemployed	299	17.7	4 (1.3)	0.038
Peasant farmer/casual	934	55.3	20 (2.1)	
Small scale business	367	21.7	2 (0.5)	
Skilled/professional	90	5.3	4 (4.4)	
Place of delivery^a^ (*N* = 1724)				
Medical facility	1505	87.3	1.5 (22)	0.025
Home	219	12.7	4.1 (9)	
Breast feeding type^a^ (*N* = 1219)				
On demand	1160	95.2	0.9 (11)	0.027
Time table	59	4.8	5.1 (3)	

^**a**^variables with missing information

Rubella sero-prevalence was statistically different depending upon urban versus rural residences (*p* = 0.018). Mother’s occupation (*p* = 0.038), place of delivery (*p* = 0.025), and breast feeding type (*p* = 0.027) were also statistically significant epidemiologic factors. Children delivered at home had increased odds of rubella as compared to those delivered at health facilities (OR 2.6 95% CI: 1.2,5.8), Children who were breastfed on time table had increased odds as compared to those breastfed on demand (OR 5.6, 95% CI: 1.5, 20.6) (Table 2). We did not observe statistically significant variation in rubella sero-prevalence depending upon maternal age, maternal education, parity and the number of live children.

**Table 2:** Factors associated with rubella sero-prevalence among children aged 7–36 months

Exposure variables	Crude OR	*P*-value	95% C. I.		Adjusted OR	*P*-value	95% C. I.	
			Lower	Upper			Lower	Upper
District								
Same	1	-	-	-	1	-	-	-
Mwanga	0.404	0.153	0.116	1.401	0.525	0.337	0.141	1.956
Rombo	0.295	0.011	0.115	0.758	0.393	0.072	0.142	1.088
Moshi District	0.236	0.003	0.092	0.604	0.268	0.015	0.093	0.776
Residence								
Rural	1	-	-	-	1	-	-	-
Urban	0.304	0.051	0.092	1.005	2.497	0.203	0.611	10.199
Mother’s occupation								
Skilled	1	-	-	-	1	-	-	-
Unemployed	0.292	0.086	0.071	1.190	0.613	0.512	0.142	2.647
Peasant farmer	0.470	0.178	0.157	1.408	0.412	0.345	0.065	2.601
Small business & unskilled	0.118	0.014	0.021	0.654	0.855	0.896	0.081	9.014
Place of delivery								
Medical facility	1	-	-	-	1	-	-	-
Home	2.648	0.015	1.210	5.797	2.037	0.378	0.419	9.901
Breast feeding								
On demand	1	-	-	-	1	-	-	-
Time table	5.596	0.010	1.518	20.625	5.179	0.052	0.989	27.136

*OR* Odds ratio, *CI* Confidence interval

In Multivariate analysis Moshi district had significantly lower odds of rubella sero-prevalence (OR 0.3, 95% CI: 0.1, 0.8) (Table 2).

## Discussion

This is the first community based study, characterizing rubella epidemiology in Kilimanjaro region, Tanzania. The study found a low overall rubella sero-prevalence in the region (1.8%), signifying low natural immunity to rubella, rendering children to a high susceptibility (98.2%) for infection in the event of an outbreak of wild rubella virus. This low level of natural immunity warrants the inclusion of the rubella vaccine in routine childhood immunization in this region. This finding is similar to other pre-vaccine rubella epidemiologic studies, documenting the peak age of infection among children of 5–9 years [22]. Rubella epidemics tend to repeat every 5–9 years, which can partly explain the low sero-prevalence in young children born prior to an epidemic period [24]. The results are similar with findings from Bangladesh where Sultana et al. reported a lack of protective antibodies against rubella among children 3 months to 5 years, however demonstrated increasing rubella sero-prevalence with age to 71% at >10–15 years [26].

Infants below 7 months were not included in the analysis as all had low antibodies levels below the test cut-off threshold. The low antibody titers can be attributed to non–immune mothers, a finding reported by Mwambe and colleagues in a study of pregnant women in Mwanza, Tanzania [32]. Early waning of passive antibodies, is another possible explanation for low level of antibodies in children below 7 months, a finding reported by Manirakiza and colleagues in Central African Republic [27], where maternal rubella antibodies sero-prevalence rates were >45% among infants 0–3 months, decreasing to >10% at 4–6 months and finally to zero at 7–12 months. The observed waning of passive antibodies at 7 months, may explain the increasing susceptibility, leading to increased rubella infection for infants above 6 months in our study [33].

Our findings provide important information in choosing the appropriate vaccination age. In Tanzania, rubella vaccine is combined with the measles vaccine (MR), and since rubella is a mild disease, measles immunology and epidemiology determines the optimal timing for MR vaccination. Currently, MR is administered in two doses, the first dose at 9–12 months, and the second dose at 15–18 months [34]. Our findings on rubella Sero-epidemiology, confirms that, the schedule for MR vaccine at this age will be an effective timing, as 98% of the children have no immunity against rubella infection.

This study found an association between rubella sero-prevalence and district of residence. Other test variables including: gender, age, rural versus urban residence, mother’s occupation, place of delivery and breast feeding type were determined to be statistically insignificant factors in our analysis [24, 27]; however, as reported elsewhere, these factors were found to be significant factors for pre-vaccine era rubella epidemiology [26]. Manirakiza and colleagues in a Central African study reported no gender differences in rubella sero-prevalence; however, they did report an increase in rubella sero-prevalence with increasing age [27]. Similarly, Ki and colleagues reported an increase in sero-prevalence with increasing child age in a study among Korean children [35]. A study in Mwanza, Tanzania conducted by Mirambo et al., found that, rural residence and child age were statistically significant associated factors for rubella infection [24]. Higher risk of rubella infection in rural areas was also reported in a recent Kenyan study, by Kombich and colleagues, where lower social economic status and age (above 7 years) were other associated risks for rubella infection [36]. The differences in sero-prevalence between urban and rural settings points to special considerations for targeted vaccination campaigns to specific high risk communities.

## Conclusions

The data presented in this study were obtained before introduction of rubella vaccine in Kilimanjaro region, Tanzania. The study findings of low natural immunity to rubella among children in the age group of 7 to 36 months is significant, as well as the increase of rubella prevalence with age, which poses a risk for rubella epidemics and increase in CRS. These findings justify the need for introduction of rubella immunization to eliminate rubella transmission. A successful immunization strategy in Tanzania demands further evidence-based guidance from additional epidemiologic and immunologic studies performed across this region.

We recommended further epidemiologic studies to establish the burden and risk factors for rubella infection and CRS, as well as to establish geographical immunity gaps. Post-rubella vaccine introduction studies are necessary to characterize current vaccine coverage, potential barriers to intervention, safety and efficacy of RCVs, with special focus on disadvantaged rural populations.

## Additional file

Additional file 1: Past and current Childhood immunization schedule in Tanzania. (DOCX 12 kb)

## Abbreviations

CRS: Congenital rubella syndrome
DBS: Dried blood spot
ELISA: Enzyme linked immunosorbent assay
HIV: Human immunodeficiency virus
IgG: Immunoglobulin G
KCMC: Kilimanjaro Christian Medical Centre
MMRV: Measles Mumps Rubella Varicella
OD: Optical density
PCR: Polymerase chain reaction
RCV: Rubella containing vaccines
REK: Regional Committee for Medical & Health Research Ethics
WHO: World Health Organization
