# Supplementary material for: Rubella sero-prevalence among children in Kilimanjaro region: a community based study prior to the introduction of rubella vaccine in Tanzania
Source: Ital J Pediatr. 2017 Jul 21;43:63. doi: 10.1186/s13052-017-0379-3 (PMC5521071; doi:10.1186/s13052-017-0379-3)
Supplement: Additional file 1: — Past and current Childhood immunization schedule in Tanzania. (DOCX 12 kb) [file 13052_2017_379_MOESM1_ESM.docx]

**Childhood Immunization schedule of 2010 and the current one in Tanzania.**

| Antigen | 2010 schedule | Current Schedule |
| --- | --- | --- |
| OPV0 | At birth up to 14 days | At birth up to 14 days |
| BCG | At birth or first contact | At birth or first contact |
| OPV1, DTP-HepB-Hib1 | 4 Weeks | 6 weeks |
| OPV2, DTP-HepB-Hib 2 | 8 Weeks | 10 weeks |
| OPV3, DTP-HepB-Hib 3 | 12 Weeks | 14 weeks |
| Measles | 9 Months |  |
| PCV1 |  | 6 weeks |
| PCV2 |  | 10 weeks |
| PCV3 |  | 14 weeks |
| ROTAVIRUS |  | 6 and 10 weeks |
| MEASLES1 | 9 months |  |
| MEASLES2 | 18 months |  |
| MEASLES & RUBELLA 1* |  | 9 months |
| MEASLES & RUBELLA2* |  | 18 months |

*Rubella Containing Vaccine was introduced in 2014.

OPV= Oral Polio Vaccine, DTP = Diphtheria, Tetanus Pertussis; HepB= Hepatitis B virus; Hib; Haemophylus influenza type b; PCV= Pneumococcal conjugate vaccine.
